# Supplementary material for: Maternal TDP-43 interacts with RNA Pol II and regulates zygotic genome activation
Source: Nat Commun. 2023 Jul 17;14:4275. doi: 10.1038/s41467-023-39924-1 (PMC10352294; doi:10.1038/s41467-023-39924-1)

Supplementary figures and their legends

Maternal TDP-43 regulates zygotic genome activation through RNA Pol II

Xiaoqing Nie, Qianhua Xu, Chengpeng Xu, Fengling Chen, Qizhi Wang, Dandan Qin, Rui Wang, Zheng Gao, Xukun Lu, Xinai Yang, Yu Wu, Chen Gu, Wei Xie, Lei Li

Correspondence: xiewei121@tsinghua.edu.cn, lil@ioz.ac.cn

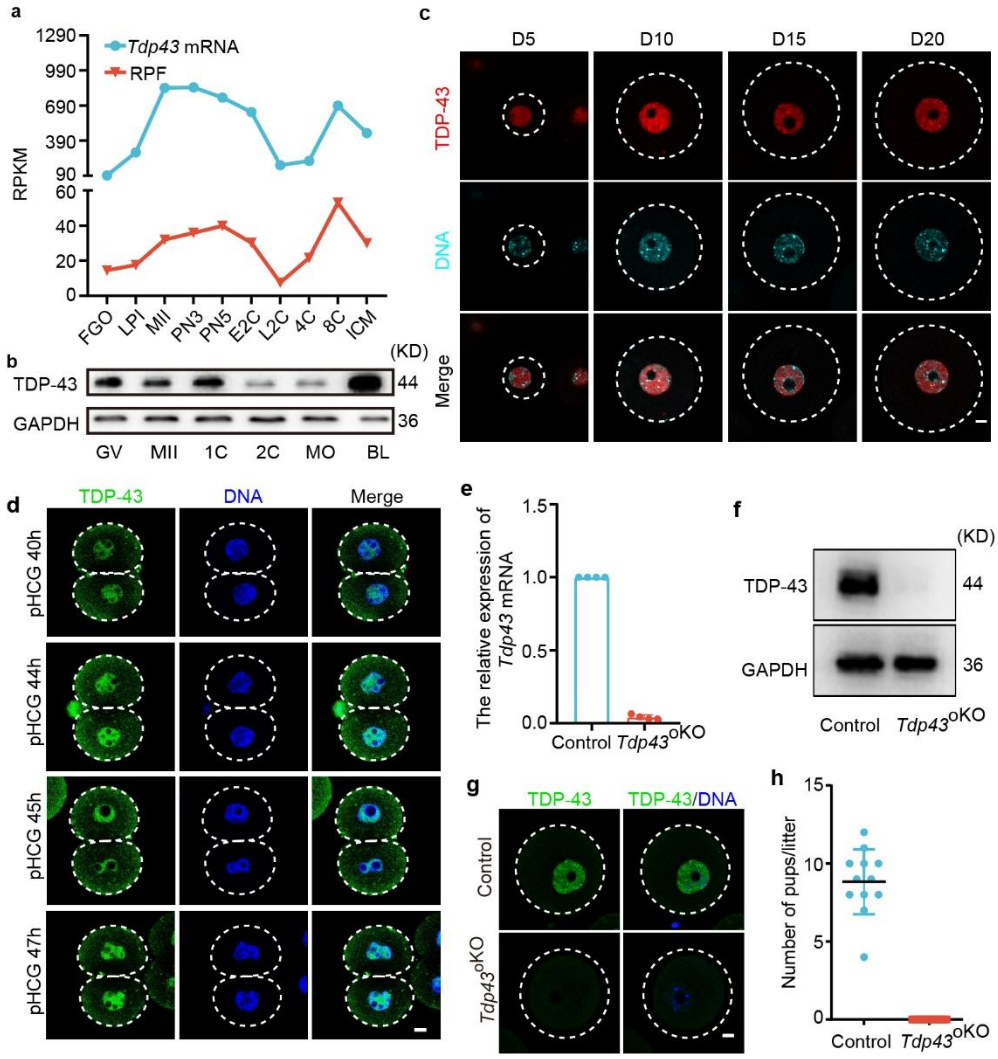

Supplementary Fig. 1 The expression of TDP-43 in oocytes and early embryos and knockout efficiency. a, The dynamics of mRNA (mRNA-seq signal) and RPF (Ribo-seq signal, represent the efficiency of translation) of TDP-43 in mouse oocytes and early embryos. the data are presented as the mean of RPKM from two repeats of Ref. (Xiong, et al.,2022). RPKM, reads per kilobase of bin per million mapped reads; FGO, LPI, and MII, full-grown, late prometaphase I and MII oocytes; PN3 and PN5, PN3 and PN5 stage of early 1-cell embryos; E2C and L2C, early and late 2-cell embryos; 4C and 8C, 4-, and 8-cell embryos; ICM, inner cell mass. **b**, Western blot showing the expression of TDP-43 in mouse oocytes and early embryos. GV and MII, GV and MII oocytes; 1C and 2C, 1- and 2-cell embryos; MO, morulae; and BL, blastocysts. **c**, Immunostaining of TDP-43 for mouse oocytes at different stages. These oocytes were isolated from mouse ovaries at the day-fifth (D5), -tenth (D10), -fifteenth (D15), -twentieth (D20) after birth. Scale bar, 10 μm. **d**, Immunostaining of TDP-43 for mouse 2-cell embryos at different time points. pHCG, post HCG injection. h, hour. Scale bar, 10 μm. **e**,**f**,**g**, The deletion efficiency of *Tdp43* at the levels of mRNA (**e**) and protein as shown with

Western blotting (**f**) and immunostaining (**g**). The dots represent the number of four independent experiments, and the data are presented as mean ± SD (standard derivation) in (**e**). Scale bar, 10 μm. **h**, The number of pups per litter from the control (n=8) and *Tdp43*^{oKO} females (n=8). Black line, the mean of pups. Error bars, SD.

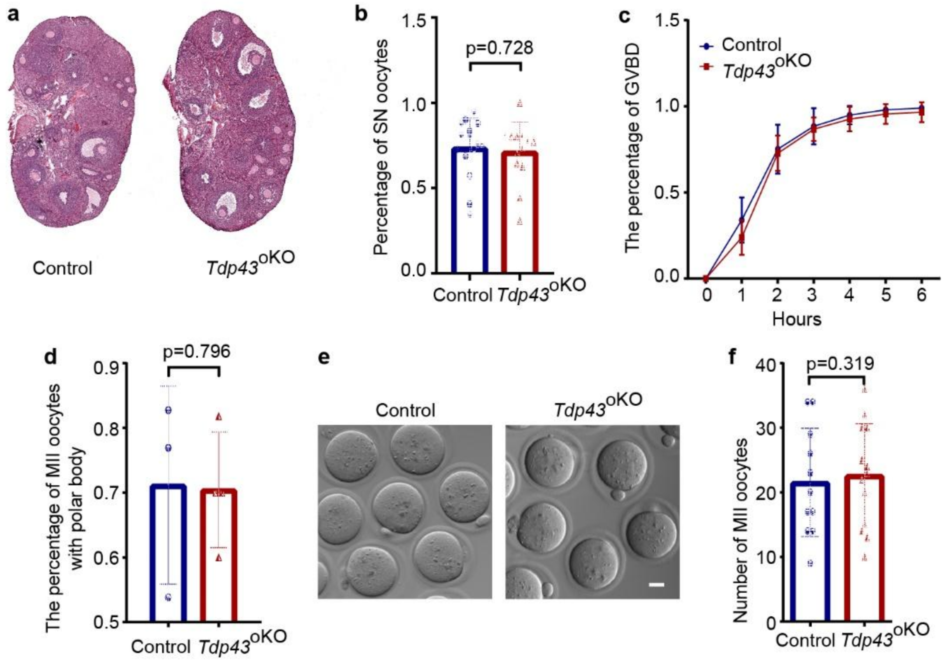

Supplementary Fig. 2 *Tdp43* is dispensable for oocyte maturation and ovulation. **a**, The representative ovary section from 8-week control and *Tdp43*^{oKO} females. **b**, The rate of SN oocytes. Mouse GV oocytes were collected from control (n=15) and *Tdp43*^{oKO} (n=13) females after 46 hours of PMSG injection and were stained with DAPI. NSN (non-surrounded nucleolus) and SN (surrounded nucleolus) oocytes were counted. The rate of SN oocytes was calculated and are presented as mean ± SD (standard derivation). P-value were calculated through two-tailed Student's t-test. **c**, GVBD rate of the GV oocytes in control and *Tdp43*^{oKO} mice. GV oocytes were isolated from control and *Tdp43*^{oKO} females after 46 hours of PMSG injection. The data were from seven independent experiments (at least 29 oocytes per experiment). Solid circles or quadrates, mean. Error bars, SD. **d**, Percentage of the MII oocytes with polar body. MII oocytes were isolated from control and *Tdp43*^{oKO} females after 13 hours of HCG injection. The data were from three independent experiments (at least 27 oocytes per experiment) and are presented as mean ± SD. P-value were calculated through two-tailed Student's t-test**.** **e**, Representative DIC images of MII oocytes from control and *Tdp43*^{oKO} females. Scale bar, 20 μm. **f**, Number of the MII oocytes from control (n=11) and *Tdp43*^{oKO} (n=13) mice. MII oocytes were collected from control and *Tdp43*^{oKO} females after 13 hours of HCG injection. The data are presented as mean ± SD. P-value were calculated through two-tailed Student's t-test**.** The dots represent the number of females used for the experiments.

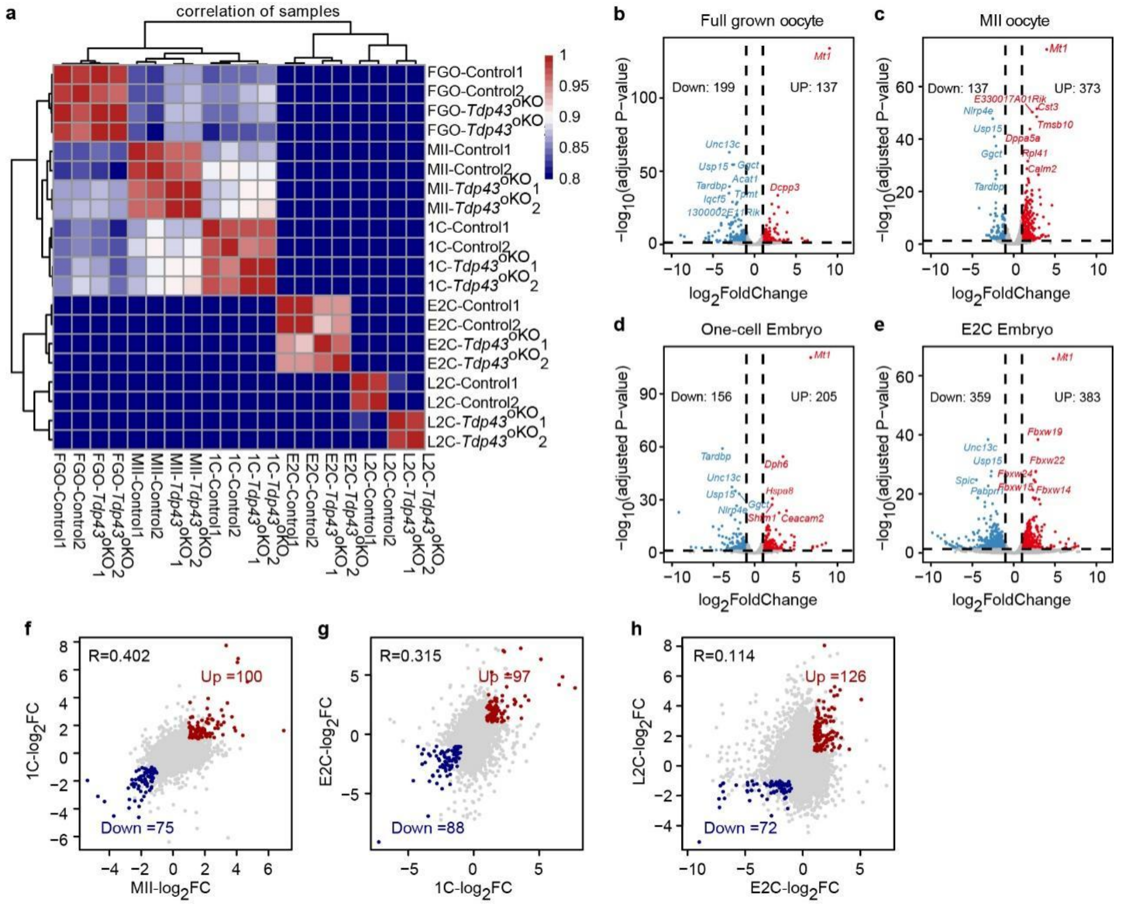

Supplementary Fig. 3 The effect of maternal TDP-43 knockout on the transcriptomes of mouse oocytes and embryos. a, Heat map showing the repeatability and correlations among control and *Tdp43*^{oKO} oocytes and embryos. FGO and MII, full-grown and MII oocytes; 1C, late 1-cell embryos; E2C, early 2-cell embryos; L2C, late 2-cell embryos. **b**,**c**,**d**,**e**, Volcano plots showing the fold change and adjust P-value of different expression genes (DEGs) between control and *Tdp43*^{oKO} in FGO (**b**), MII (**c**), 1C (**d**), and E2C (**e**). The numbers of genes with significantly up- and down-regulated expression in *Tdp43*^{oKO} were identified through compared with the control (adjust P-value ≤ 0.05; fold change ≥ 2). P-values were calculated by Wald significance tests and adjusted with Benjamini and Hochberg method with DESeq2. **f**,**g**,**h**, Scatter plot of Pearson correlation showing fold-change ($\log_2^{oKO/control} = 1$) of gene expression between MII and 1C (**f**), 1C and E2C (**g**), as well as 1C and L2C (**h**). Red and blue dots, the shared genes with Up (up-regulated) or Down (down-regulated) expression.

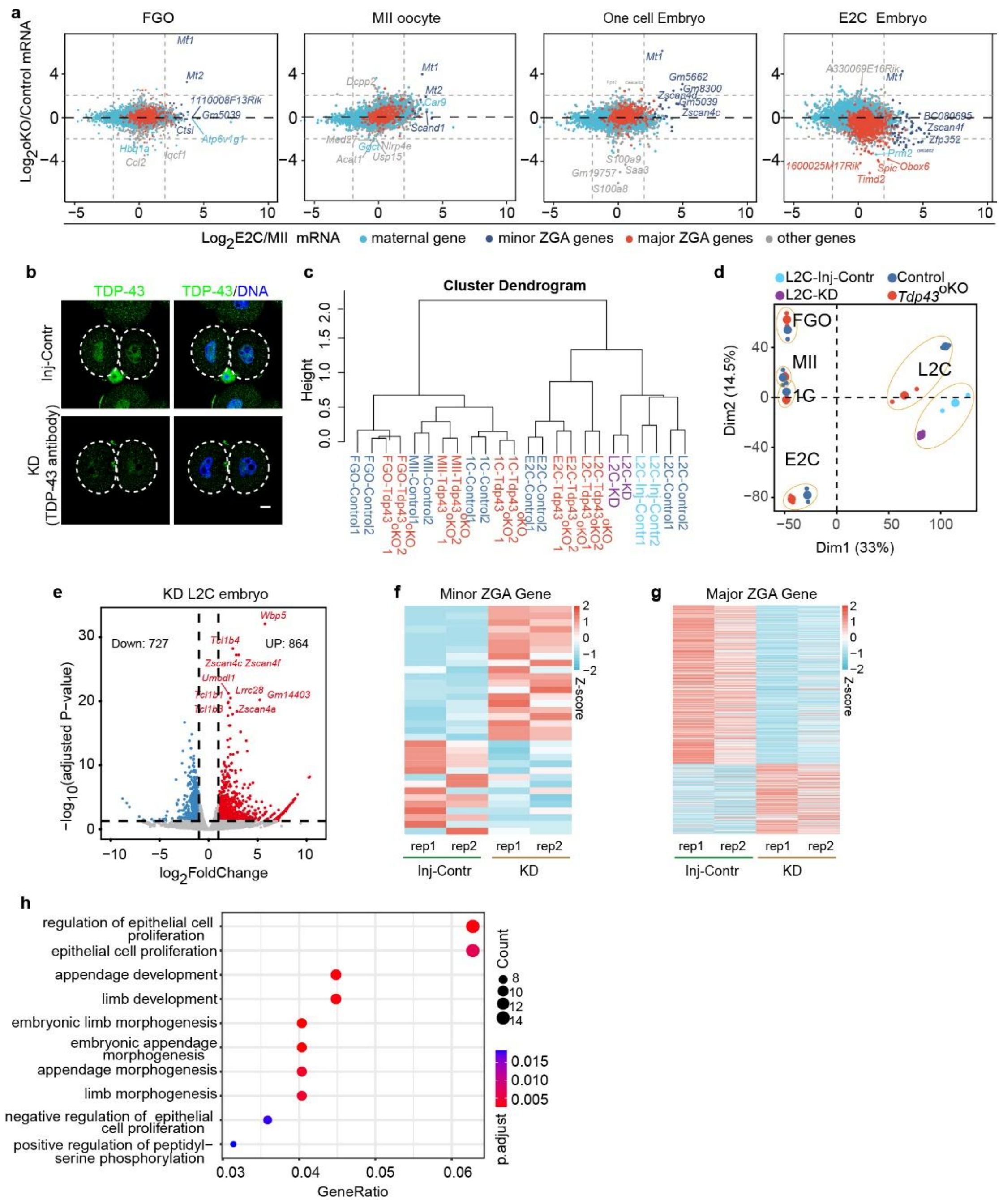

Supplementary Fig. 4 The effect of TDP-43 knockout and knockdown on mouse ZGA. a, Scatter plot showing mRNA fold-change (log2 ratio) between Control and *Tdp43*^{oKO} group (oKO) in L2C (Y-axis), as well as mRNA fold-change between E2C and MII (X-axis) in FGO, MII, 1C and E2C. Green, blue, red and grey dots delineate maternal, minor, major and other genes, respectively. **b**, Immunostaining of TDP-43 in KD (knockdown by the Trim-away with TDP-43 antibody and *Trim21* mRNA, Materials and Methods) and Inj contr (injection control with IgG and *Trim21* mRNA) L2C embryos. Scale bar, 10 μm. **c**,**d**, Principal component analysis (**c**) and Hierarchical clustering analysis (**d**) for RNA-seq data among FGO, MII, 1C, E2C and L2C

from control and *Tdp43*^{oKO} females, as well as TDP-43 KD and Inj-contr L2C embryos. **e**, Volcano plot showing different expression genes (DEGs) between KD and Inj-contr L2C groups. 727 (Down) and 864 (Up) represent the number of genes with significantly up- and down-regulated expression that were identified in KD when compared with the Inj-control (adjust P-value ≤ 0.05; fold change ≥ 2). P-values were calculated by Wald significance tests and adjusted with Benjamini and Hochberg method with DESeq2, Inj-contr, the control for KD. **f**,**g**, Heat map showing the expression of minor ZGA genes (**f**) and major ZGA genes (**g**) in Inj-contr and KD embryos. Rep, repeat sample. **h**, GO terms of the up-regulated genes affected in both *Tdp43*^{oKO} (Figure 2b) and KD L2C embryos (Figure S 4e). P-values were calculated through one-sided hypergeometric test. The size of dots represents the number of specific genes.

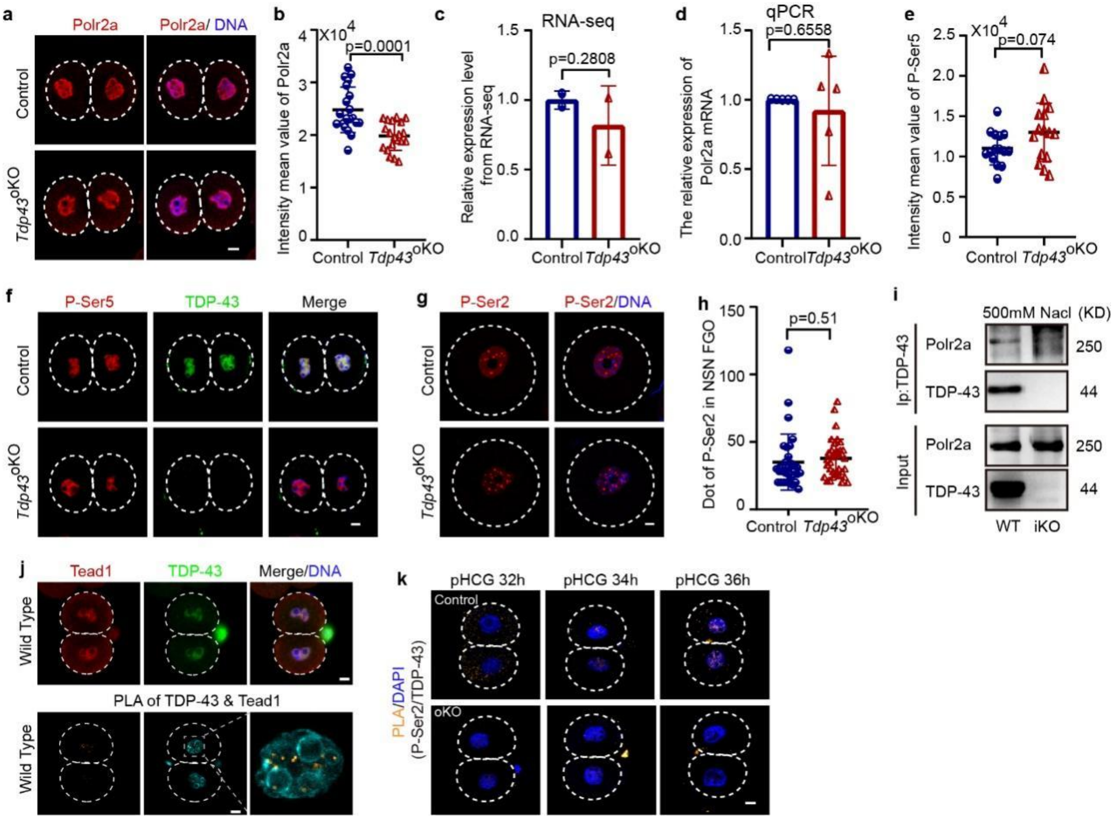

Supplementary Fig. 5 The relationship between TDP-43 and Pol II. **a**, Polr2a staining in control and *Tdp43*^{oKO} L2C embryos. Scale bar, 10 μm. **b**, Intensity of the Polr2a staining in control (n=20) and *Tdp43*^{oKO} (n=20) L2C embryos. Black line, mean. Error bars, SD. P-value is analyzed through two-tailed Student's t-test. **c**,**d**, The relative expression of Polr2a mRNA (**c**, RNA-seq from two independent experiments; **d**, quantitative RT-PCR from five independent experiments) in Control and *Tdp43*^{oKO} L2C. Control was normalized as 1.The data are presented as mean ± SD. P-value was from the two-tailed Student's t-test. **e**, The intensity of P-Ser5 staining in control (n=15) and *Tdp43*^{oKO} (n=15) L2C embryos. Black line, mean. Error bars, SD. P-value was analyzed through two-tailed Student t-test**.** **f**, P-ser5 staining in control and *Tdp43*^{oKO} L2C embryos. Scale bar, 10 μm. **g**, P-ser2 staining in NSN GV oocytes from control and *Tdp43*^{oKO} females. Scale bar, 10 μm. **h**, Number of the foci of P-ser2 staining in NSN GV control (n=32) and *Tdp43*^{oKO} (n=36) oocytes from control (n=3) and *Tdp43*^{oKO} (n=3) females. P-value was calculated through two-tailed Student t-test. Black line, mean. Error bars, SD. **i,** Polr2a was pulled down by TDP-43 antibody in mouse ESC lysis under the stringent condition (washed with 500 mM NaCl). The iKO ESCs were treated with Tamoxifen for 72 hours (TDP-43 KO) and used for the negative control. **j**, Immunostaining (upper panel) for transcription factor Tead1 and PLA (lower panel) with Tead1 and TDP-43 antibody in WT 2-cell embryos (n≥15). The TDP-43 antibody was same as in Fig. 3d-f (lower panel). Scale bar, 10 μm. The PLA with TDP-43 and Tead1 antibody was performed as additional negative

control. **k**, PLA of TDP-43 and P-Ser2 in control and *Tdp43*^{oKO} (oKO) E2C embryos at different time points (n≥15). Scale bar, 10 μm.

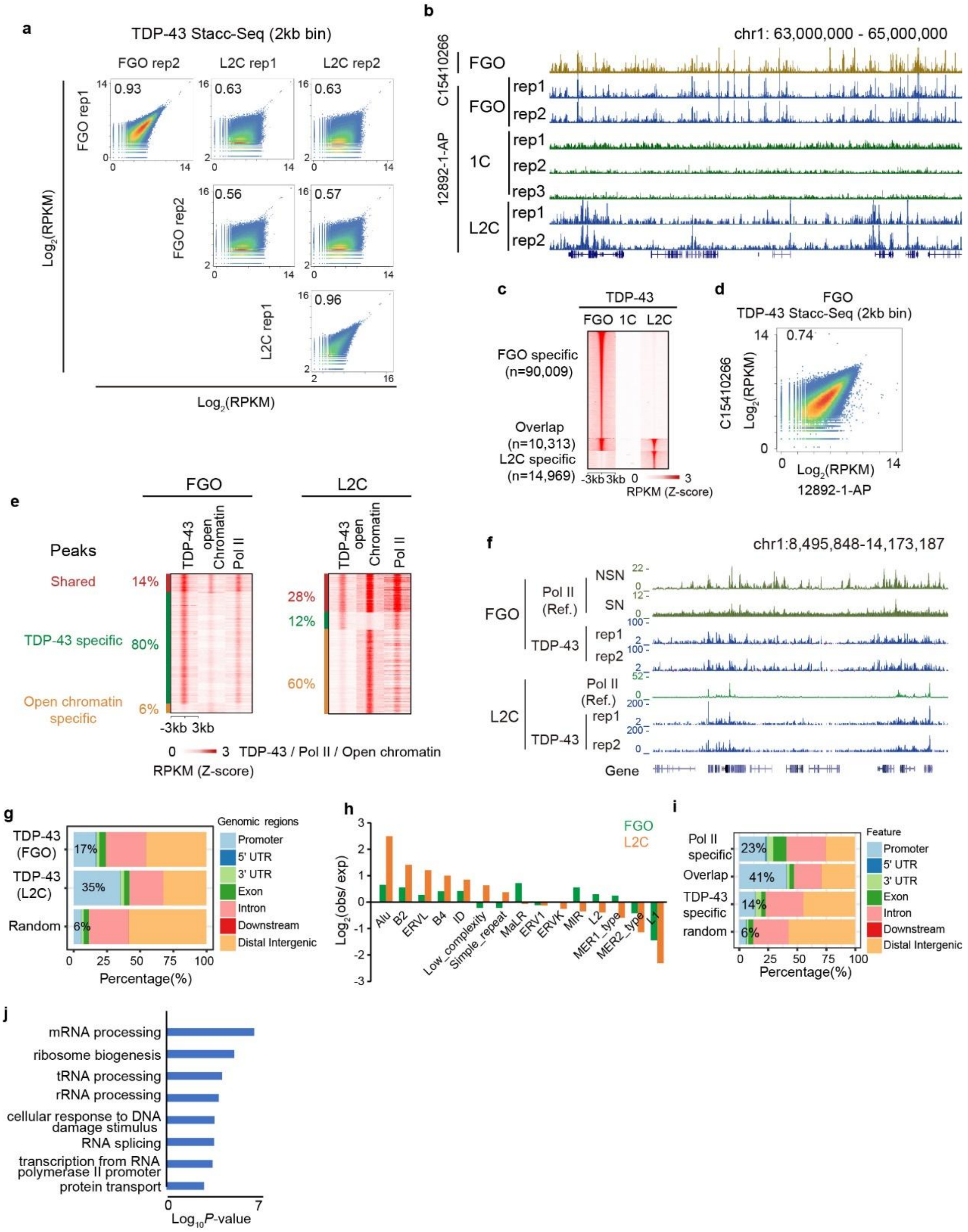

Supplementary Fig. 6 Validation of TDP-43 Stacc–seq in FGOs and early embryos. **a**, Scatter plots comparing genome-wide TDP-43 enrichment (2-kb bins) between FGO (full-grown oocytes) and L2C (late 2-cell embryo) replicates for the Stacc-seq data. Pearson's correlation coefficients were shown on the top-left panel. RPKM, reads per kilobase of bin per million mapped reads. **b**, Snapshot showing the TDP-43 signals in FGO, 1C (1-cell) and L2C embryos identified with two antibodies against for TDP-43 (C15410266 and 12892-1-AP, Material and Methods). **c**, Heat map showing

the enrichment of TDP-43 in wild type FGO, 1C and L2C. **d**, Scatter plot comparing the genome-wide TDP-43 enrichment (2-kb bins) in FGO identified by the two different antibodies as (b). Pearson's correlation coefficients were shown on the top-left panel. **e**, Heat maps showing the enrichment of TDP-43, Pol II (Ref., Liu, et al., 2020) and open chromatin (Ref., Inoue et al., 2017, Wu, J. et al., 2016) at the shared, TDP-43-specific and open chromatin-specific peaks in FGO and L2C. Z-score was normalized. **f**, Snapshot showing Pol II and TDP-43 engagement at the same loci occupation in Ref oocytes and embryos (Ref, Liu, et al., 2020), as well as normal GV oocytes and L2C. **g**, Percentage stacking bar plot showing TDP-43 occupancy at genome segmentation in FGO and L2C. **h**, Histogram showing TDP-43 enrichment at repeat subfamilies in FGO and L2C compared with those in random sequence. **i**, Percentage stacking bar plot showing TDP-43 and Pol II occupancy at genome segmentation in L2C embryos. Random shuffled peaks were used as the control. **j**, GO terms of TDP-43 and Pol II co-occupying ZGA genes in L2C embryos. P-values (≤ 0.001) were calculated with two-sided Fisher's exact test.

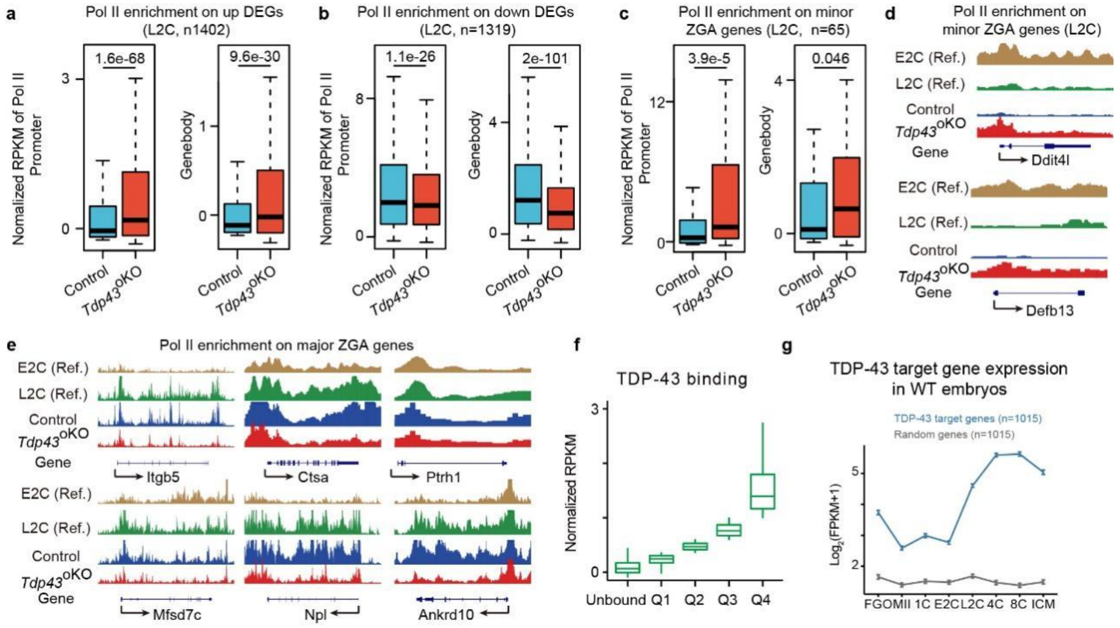

Supplementary Fig. 7 TDP-43 Disruption affects the occupancy of Pol II in L2C embryos. **a**,**b**,**c**, Box plots showing the enrichment of Pol II at promoters (left) and bodies (right) of the identified up-DEGs (**a**), down-DEGs (**b**) and minor ZGA genes (**c**) in L2C. Upper and lower line of the box represent the 25th and 75th percentiles. Whiskers show 1.5X interquartile range. Central lines, the medians. All tests were run with the two-sided Wilcoxon rank-sum**.** **d**,**e**, Snapshot showing Pol II enrichment at representative minor ZGA genes (**d**) and major ZGA genes (**e**) in Ref E2C and L2C (Liu, et al., 2020), as well as control and *Tdp43*^{oKO} L2C. **f**, The strength of TDP-43 binding were classified into five categories according to their binding enrichment of TDP-43 at all genes (Unbound,15325 genes; Q1, 1843 genes; Q2, 1847 genes; Q3,1850 genes; Q4, 1848 genes). Upper and lower line of the box represent the 25th and 75th percentiles. Whiskers show 1.5X interquartile range. Central lines, the medians. **g**, The expression of TDP-43 targeting genes of L2C in mouse pre-implantation embryos. The data of RNA-seq were obtained from the published resource (Zhang et al., 2016). The data were shown as mean ± SE. FGO, full-grown oocyte; MII, MII oocyte; 1-8C, 1-8 cell embryo; ICM, inner cell mass.

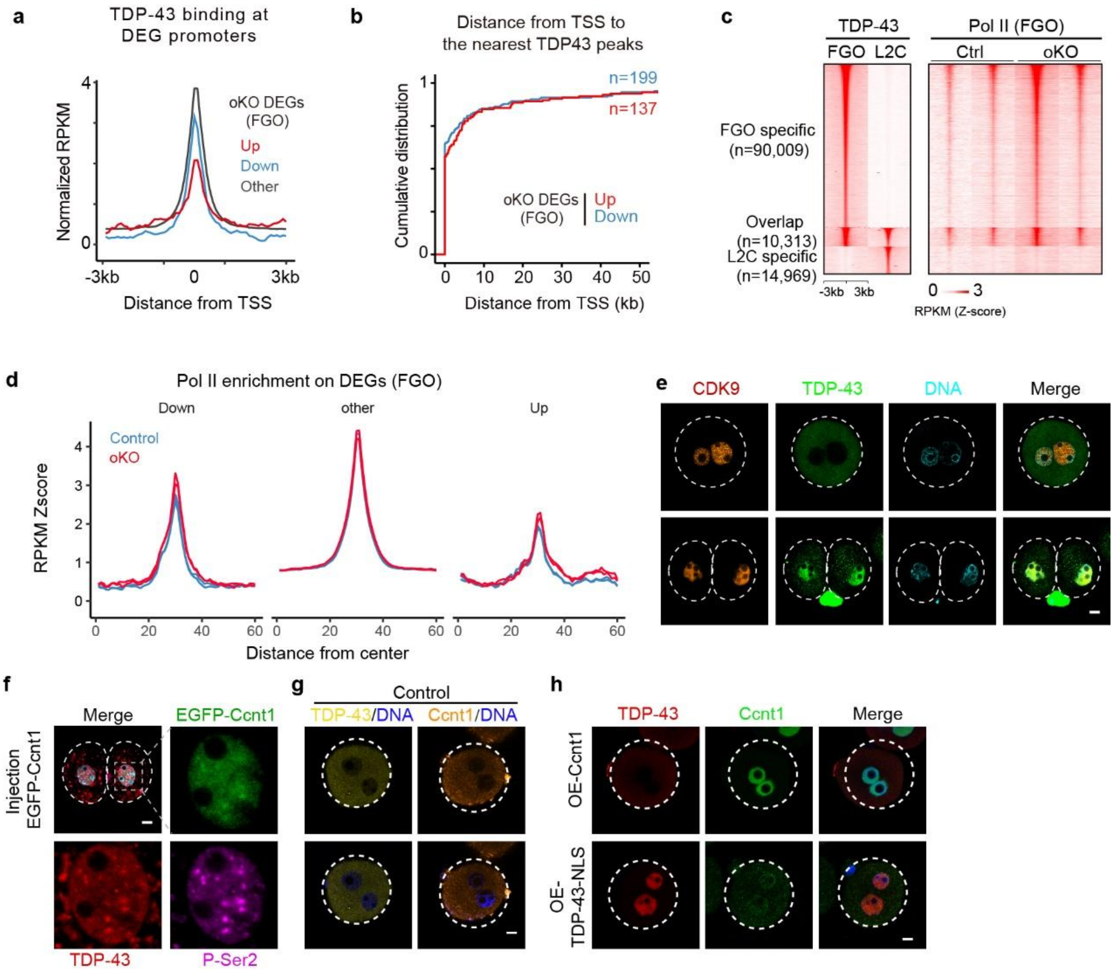

Supplementary Fig. 8 TDP-43 binding and the gene expression. a, Enrichment of TDP-43 at the promoter of DEGs (different expression genes, up- and down-regulated genes) in control and *Tdp43*^{oKO} FGO. **b**, Distance from the TSS of DEGs to the nearest TDP-43 peak in FGO. TSS, transcription start sites. **c**, Heat maps showing the enrichment of TDP-43 and Pol II at the FGO and L2C specific peaks, as well as the overlapped peaks in FGO and L2C. TDP-43 enrichment was shown in the left panel. Pol II enrichment in FGO was shown in the right panel. Ctrl, control; oKO, *Tdp43*^{oKO}. **d**, Pol II enrichment at the DEGs of FGO. **e**, Staining of CDK9 and TDP-43 in normal 1- and 2-cell embryos. Scale bar, 10 μm. **f**, Co-localization of Cyclin T1, P-Ser2 and TDP-43. normal 1-cell embryos were injected with *Ccnt1-EGFP* mRNA, and were cultured until L2C (pHCG 48h). The cultured L2C embryos were fixed with 4% PFA and 0.5% Triton X100 for 25 mins, and were stained with the P-Ser2 and TDP-43 antibody. Scale bar, 10 μm. **g**, The staining of TDP-43 and Ccnt1 in wild type mouse 1-cell embryo. Scale bar, 10 μm. **h**, The staining of endogenous TDP-43 and Ccnt1 in the injection mouse 1-cell embryos with the over-expression of Ccnt1-EGFP and NLS-TDP-43-mCherry. Mouse early 1-cell embryos were injected the mRNA of *Ccnt1-EGFP* or

NLS-Tdp43-mCherry and cultured for the expression of EGFP and mCherry. These 1-cell embryos were fixed and stained with the antibodies against for TDP43 and Ccnt1. NLS, nuclear localization signal. Scale bar, 10 μm.

Source data for Western blots in supplementary materials

Supplementary Fig. 1b Western blot of TDP-43 and GAPDH with 50 oocytes or early embryos

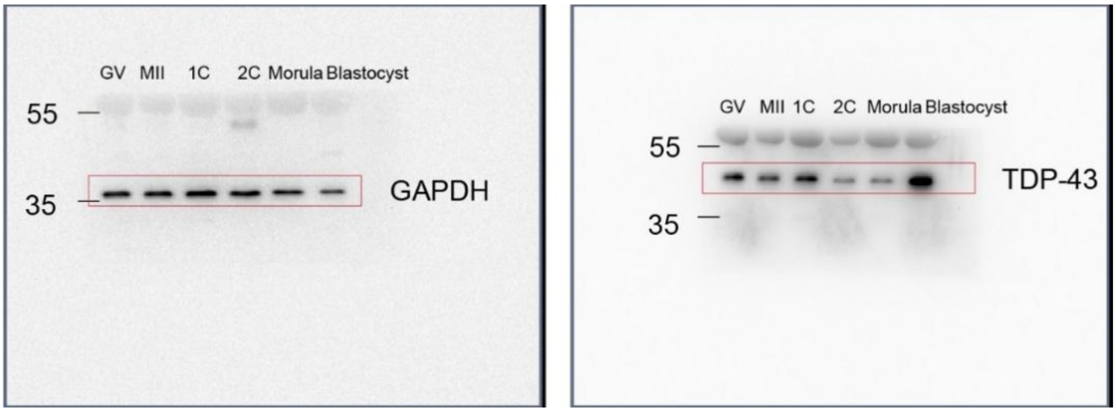

Supplementary Fig. 1f Western blot of TDP-43 and GAPDH with 50 oocytes or early embryos from control and oKO mice

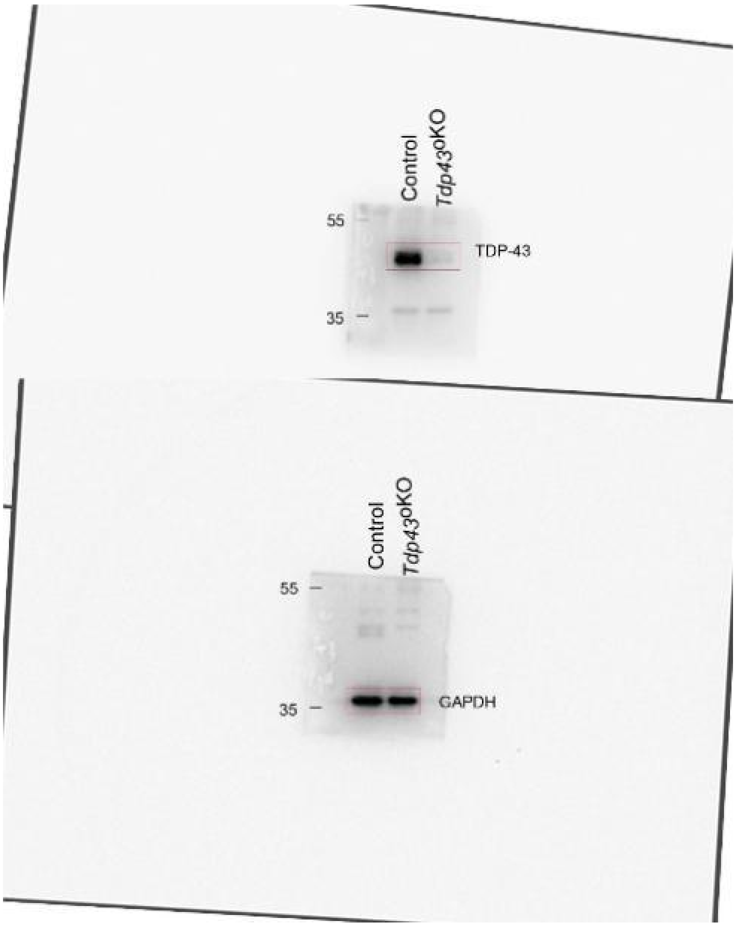

Supplementary Fig. 5i IP of TDP-43 being washed with NaCl

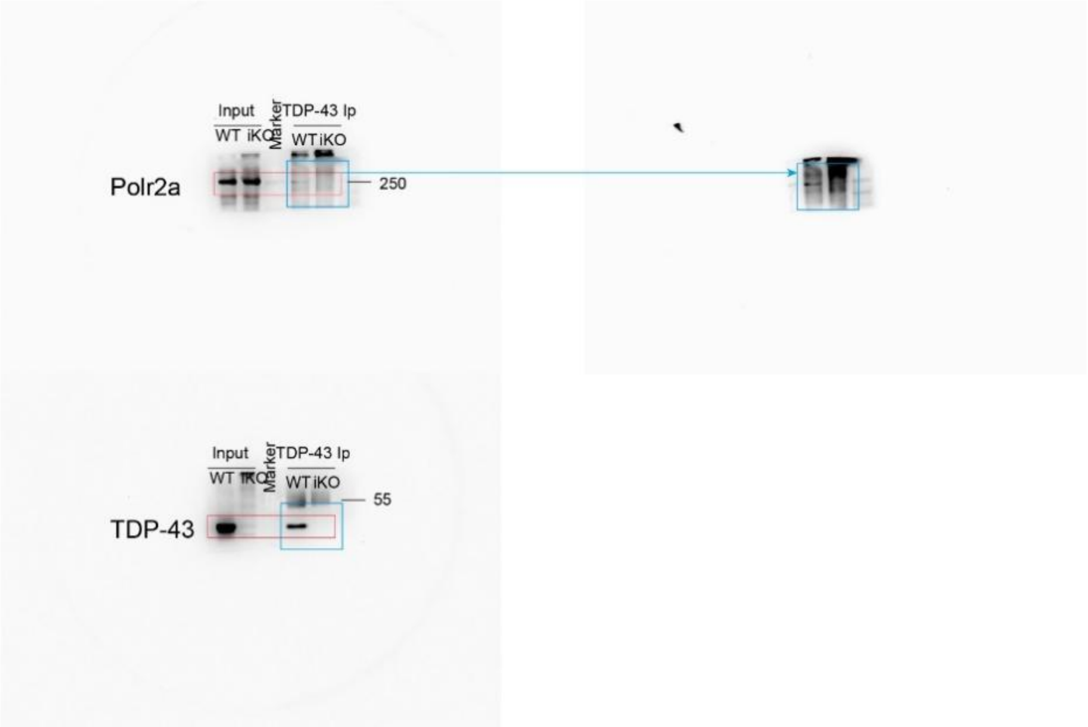

Supplement: Supplementary file 1 — Supplementary Information [file 41467_2023_39924_MOESM1_ESM.pdf]
