# Supplementary material for: Maternal TDP-43 interacts with RNA Pol II and regulates zygotic genome activation
Source: Nat Commun. 2023 Jul 17;14:4275. doi: 10.1038/s41467-023-39924-1 (PMC10352294; doi:10.1038/s41467-023-39924-1)
Supplement: Supplementary file 3 — Reporting Summary [file 41467_2023_39924_MOESM3_ESM.pdf]

nature portfolio

Corresponding author(s): Lei Li

Last updated by author(s): 2023-6-23

Reporting Summary

Nature Portfolio wishes to improve the reproducibility of the work that we publish. This form provides structure for consistency and transparency in reporting. For further information on Nature Portfolio policies, see our Editorial Policies and the Editorial Policy Checklist.

Statistics

For all statistical analyses, confirm that the following items are present in the figure legend, table legend, main text, or Methods section.

n/a	Confirmed	
☐	☒	The exact sample size (*n*) for each experimental group/condition, given as a discrete number and unit of measurement
☐	☒	A statement on whether measurements were taken from distinct samples or whether the same sample was measured repeatedly
☐	☒	The statistical test(s) used AND whether they are one- or two-sided<br>*Only common tests should be described solely by name; describe more complex techniques in the Methods section.*
☒	☐	A description of all covariates tested
☐	☒	A description of any assumptions or corrections, such as tests of normality and adjustment for multiple comparisons
☐	☒	A full description of the statistical parameters including central tendency (e.g. means) or other basic estimates (e.g. regression coefficient) AND variation (e.g. standard deviation) or associated estimates of uncertainty (e.g. confidence intervals)
☐	☒	For null hypothesis testing, the test statistic (e.g. *F*, *t*, *r*) with confidence intervals, effect sizes, degrees of freedom and *P* value noted<br>*Give P values as exact values whenever suitable.*
☒	☐	For Bayesian analysis, information on the choice of priors and Markov chain Monte Carlo settings
☒	☐	For hierarchical and complex designs, identification of the appropriate level for tests and full reporting of outcomes
☐	☒	Estimates of effect sizes (e.g. Cohen's *d*, Pearson's *r*), indicating how they were calculated

Our web collection on statistics for biologists contains articles on many of the points above.

Software and code

Policy information about availability of computer code

Data collection	No special software was used.
Data analysis	HISAT2 v2.2.1, StringTie v2.1.2,HTSeq v2.2.1, DESeq2 v1.24.0, FeatureCounts v.2.0.1,DAVID v6.8, Bowtie2 v2.2.5, MACS v1.4.2, ChIPseeker v1.26.2, BEDTools v2.29.2,Cutadapt v.0.3.8,FeatureCounts v.2.0.1,factoextra v.1.0.7,clusterProfiler v.4.6.0, R. 2D v4.4.2, UCSC genome browser, graphpad prism8

For manuscripts utilizing custom algorithms or software that are central to the research but not yet described in published literature, software must be made available to editors and reviewers. We strongly encourage code deposition in a community repository (e.g. GitHub). See the Nature Portfolio guidelines for submitting code & software for further information.

Data

Policy information about availability of data

All manuscripts must include a data availability statement. This statement should provide the following information, where applicable:

- Accession codes, unique identifiers, or web links for publicly available datasets
- A description of any restrictions on data availability
- For clinical datasets or third party data, please ensure that the statement adheres to our policy

All data supporting the findings of this study are available within the paper and its Supplementary information files. All the datasets used in this study are publicly available. The raw and processed data generated in this study have been deposited in GEO with accession number GSE221985 [https://www.ncbi.nlm.nih.gov/geo/

query/acc.cgi?acc=GSE221985]. The mm9 mouse reference genome is available at https://hgdownload.soe.ucsc.edu/ goldenPath/mm9/bigZips/mm9.fa.gz. Source data are provided with this paper.

Research involving human participants, their data, or biological material

Policy information about studies with human participants or human data. See also policy information about sex, gender (identity/presentation), and sexual orientation and race, ethnicity and racism.

Reporting on sex and gender	None
Reporting on race, ethnicity, or other socially relevant groupings	None
Population characteristics	None
Recruitment	None
Ethics oversight	None

Note that full information on the approval of the study protocol must also be provided in the manuscript.

Field-specific reporting

Please select the one below that is the best fit for your research. If you are not sure, read the appropriate sections before making your selection.

☒ Life sciences ☐ Behavioural & social sciences ☐ Ecological, evolutionary & environmental sciences

For a reference copy of the document with all sections, see nature.com/documents/nr-reporting-summary-flat.pdf

Life sciences study design

All studies must disclose on these points even when the disclosure is negative.

Sample size	No statistical methods were used to predetermine sample size. For RNA-seqs, 20 oocytes or embryos were collected for each group. For stacc-seq, 150(for Pol II) or 200(for TDP-43) oocytes or embryos were obtained for each group. Determined by counting under the microscope. For immunostaining, at least 20 embryo/oocytes were observed for each investigation, and at least 5 embryos/oocytes were scanned with Zeiss fluorescence microscope for picture. Sample sizes for 2 to 3 biological replicates including all RNA-seq, TDP-43 and Pol II Stacc-seq in this study were used according to common practice in the field (Liu et al.,2020(PMID: 33116310), Zhang et al., 2016(PMID: 27626382)).
Data exclusions	There is no data that were excluded from the analyses.
Replication	Samples were collected in at least two replicates to confirm the consistency. Immunostaining and Western blots were preformed more than 3 times. All attempts at replication were successful and similar observation was made for each replicate except special mention in the response. One representative result was shown. Embryo development/MII oocytes superovulation were preformed at least 4 times. The average number were calculated with all replicates and one representative picture is shown. Replication of sequencing data was confirmed by calculating correlation and reproducibility between replicates, as shown in figure and figure legend.
Randomization	Independent repeats were performed at least twice for sequencing experiments or at least three times for other experiments. Samples for each experiment are collected randomly and independently.
Blinding	No, The conditional knockout mice have been genotyped with PCR to determine genotype. Other experimental results about biochemical or sequencing test were verified and confirmed by independent experiments by X.Q. Nie, Q.H. Xu, C.P. Xu, Z. Gao as described above with two/ three independent repeats.

Behavioural & social sciences study design

All studies must disclose on these points even when the disclosure is negative.

Study description	
Research sample	
Sampling strategy	
Data collection	

Timing

Data exclusions

Non-participation

Randomization

Ecological, evolutionary & environmental sciences study design

All studies must disclose on these points even when the disclosure is negative.

Study description

Research sample

Sampling strategy

Data collection

Timing and spatial scale

Data exclusions

Reproducibility

Randomization

Blinding

Did the study involve field work? ☐ Yes ☐ No

Field work, collection and transport

Field conditions

Location

Access & import/export

Disturbance

Reporting for specific materials, systems and methods

We require information from authors about some types of materials, experimental systems and methods used in many studies. Here, indicate whether each material, system or method listed is relevant to your study. If you are not sure if a list item applies to your research, read the appropriate section before selecting a response.

Materials & experimental systems

n/a	Involved in the study	
☐	☒	Antibodies
☐	☒	Eukaryotic cell lines
☒	☐	Palaeontology and archaeology
☐	☒	Animals and other organisms
☒	☐	Clinical data
☒	☐	Dual use research of concern
☒	☐	Plants

Methods

n/a	Involved in the study	
☐	☒	ChIP-seq
☒	☐	Flow cytometry
☒	☐	MRI-based neuroimaging

Antibodies

Antibodies used

Rabbit anti-TDP-43(Proteintech, 12892-1-AP,lot00043028&lot1 for IF1:300/WB1:1000,stacc-seq 0.5ug/time), Mouse anti-TDP-43(Santa Cruz, Sc-376532,lot #l1014, for IF1:100/WB1:1000,). Rabbit anti-TDP-43(Proteintech, 18280-1-AP, lot 00025213, for IP1:400/WB1:1000), Rabbit anti-TDP-43(diagenode C15410266,lot 43579, for stacc-seq 0.5ug/time),anti-Pol II(Active Motif 102660,for stacc-seq 0.5ug/time), Rabbit anti-Polr2a(Abclonal, A2107,lot3561768005, for IP 1:300/WB1:1000), Rabbit anti-Polr2a(affinity,DF6831,lot 83s1054 for IF1:200), Rabbit anti-Polr2a(P-Ser2)(Abcam ab5095,lot GR3195689-1&lotGR3353130-2 for IF1:250); Rabbit anti-Polr2a(P-Ser5)(Abcam, ab193467, lot GR3298885-1, for IF1:200); Rabbit anti-Ccnt1(Abcam,ab184703, lot GR250586-7, For IF1:100/WB1:800), Mouse anti-GAPDH(Proteintech,60004-1-lg,lot, 10025237 for WB1:5000), Mouse anti-Flag(Sigma Aldrich, F1804,Clone/M2, forIP1:400/WB1:1000), Mouse anti-Myc (Abmart, M20002M,lot 324572, IP1:400/WB 1:1000), Rabbit anti-CDK9(abcam, ab76320, lot GR3439647-3, for IF 1:200),Rabbit anti-Tead1(cell signaling techonology 12292S,lot 3 for IF 1:200), Cy3 donkey anti-rabbit IgG (Jackson lab, 711-165-152 ,lot 159918,1:250), Alexa Fluor 488 donkey anti-rabbit IgG(Jackson lab, 711-545-152,lot,151331,1:200), Cy5 donkey anti-rabbit IgG (Jackson lab 711-175-152, 1:400), Alexa Fluor 488 donkey anti-mouse IgG (Jackson lab 715-545-150, lot,158699, 1:200), HRP conjugated goat anti-rabbit(Jackson lab, 111-035-003,lot,111589 1:3000), HRP conjugated goat anti-mouse(Jackson lab, 115-035-003, lot,109786,1:3000)

Validation

Each primary antibody was confirmed, and the experiments used the antibodies were repeated at least three times. All antibodies were purchased from commercial companies and have been validated by the vendors, there are validation data on their manufacturers' websites (Proteintech, Santa Cruz, Abcam, Abclonal, Actif Motif,Cell Signaling, Abmart).
From the manufacturers' websites:
Rabbit anti-TDP-43(Proteintech, 12892-1-AP, for IF/WB), Rabbit mAb reacts endogenous levels of TDP-43 protein from human, mouse, rat. Western blot and immunostaining and chip analysis from various cell lines (WB: A549 cells, mouse brain tissue, HeLa cells, C6 cells, IF: HeLa cells, Neuro-2a cells) detects TDP-43 on the manufacturer's website. We have validated western blot and immunostaining with knockout oocytes.

Mouse anti-TDP-43(Santa Cruz, Sc-376532, for IF/WB) Mouse mAb reacts endogenous levels of TDP-43 protein from human, mouse, rat. Western blot and immunostaining analysis from various cell lines (WB: A-431, Hep G2 , K-562 and NIH/3T3, IF: Hep G2) detects TDP-43 on the manufacturer's website. We have validated western blot and immunostaining with knockout oocytes

Rabbit anti-TDP-43(Proteintech, 18280-1-AP, for IP1:400/WB1:1000),
Rabbit mAb reacts endogenous levels of TDP-43 protein from human, mouse, rat. Western blot and immunoprecipitation analysis of extracts from various cell lines (WB: HeLa cells, K-562 cells, IP: K-562 cells) detects TDP-43 on the manufacturer's website.

Rabbit anti-TDP-43(diagenode C15410266, for stacc-seq 0.5ug/time),
Rabbit mAb reacts endogenous levels of TDP-43 protein from human, mouse, rat. Chip-seq analysis of extracts from the K562 cells detects DNA binding by TDP-43 on the manufacturer's website.

Rabbit anti-Polr2a(Abclonal, A2107, for IP 1:300/WB1:1000),
Rabbit mAb reacts endogenous levels of Polr2a protein from human, mouse, rat. Western blot and immunoprecipitation analysis of extracts from various cell lines (WB: HeLa cells, Jurkat, mouse brain, IP: HeLa cells) detects Polr2a on the manufacturer's website.

Rabbit anti-Polr2a(Affinity,DF6831,lot 83s1054 for IF1:200),
Rabbit mAb reacts endogenous levels of Polr2a protein from human, mouse, rat. ICC analysis from rat kidney tissue detects Polr2a on the manufacturer's website.

Rabbit anti-Polr2a(P-Ser2)(Abcam ab5095, for IF1:250);
Rabbit mAb reacts endogenous levels of P-Ser2 from human, mouse, rat. IF analysis from various cell lines (HeLa cells, MCF7 and NIH-3T3 cells) detects P-Ser2 on the manufacturer's website.

Rabbit anti-Polr2a(P-Ser5)(Abcam, ab193467, for IF1:200);
Rabbit mAb reacts endogenous levels of P-Ser5 from human, mouse, rat. IF analysis from various cell lines (Rat adrenal gland pheochromocytoma cell line, (Human epithelial cell line from cervix adenocarcinoma, Mouse macrophage cell line transformed with Abelson murine leukemia virus) detects P-Ser5 on the manufacturer's website.

Rabbit anti-Ccnt1(Abcam,ab184703, For IF1:100/WB1:800),
Rabbit mAb reacts endogenous levels of Ccnt1 from human, mouse, rat. IF analysis from various cell lines (human T cell leukemia T lymphocyte and human breast adenocarcinoma epithelial cell) detects Ccnt1 on the manufacturer's website.

Mouse anti-Flag(Sigma Aldrich, F1804,Clone/M2, forIP1:400/WB1:1000),
Monoclonal antibody is produced by clone M2 and purified by affinity chromatography. The monoclonal mouse mAb reacts protein labelled by Flag. It was validated to apply to IP/WB on the manufacturer's website.

Mouse anti-Myc (Abmart, M20002M, IP1:400/WB 1:1000),
This mouse monoclonal antibody is produced by immunizing animals with a synthetic peptide (KLH-coupled) corresponding to residues 410-419 of human c-Myc (EQKLISEEDL). The mouse mAb detects over-expressed or recombinant proteins containing the Myc epitope tag. It was validated to apply to IP/WB on the manufacturer's website

Rabbit anti-CDK9(abcam, ab76320, for IF 1:200),
Rabbit mAb reacts endogenous levels of CDK9 from human, mouse, rat. IF analysis from various cell lines (Human colorectal adenocarcinoma epithelial cell and HeLa cells) detects CDK9 on the manufacturer's website.

Rabbit anti-Tead1(Cell Signaling Technology 12292S, for IF),
Rabbit mAb reacts endogenous levels of Tead1 from human, mouse, monkey. IF analysis from various cell lines (NIH:OVCAR-3 cells

and MDA-MB-453 cells) detects Tead1 on the manufacturer's website.

Eukaryotic cell lines

Policy information about cell lines and Sex and Gender in Research

Cell line source(s)	ESCs were established with the blastocysts fromTdp43flox/flox;ER-cre in Li's Lab, its male cell line. 293t cells we used was one line maintained in Li' Lab bought from Pricella(Wuhan, CL-0005).
Authentication	The ESCs were genotyped by PCR. 293t cells have been provided STR authentication profiling on the Pricella's website.
Mycoplasma contamination	All cells were tested negative for mycoplasma contamination.
Commonly misidentified lines (See ICLAC register)	None.

Palaeontology and Archaeology

Specimen provenance	
Specimen deposition	
Dating methods	

☐ Tick this box to confirm that the raw and calibrated dates are available in the paper or in Supplementary Information.

Ethics oversight	

Note that full information on the approval of the study protocol must also be provided in the manuscript.

Animals and other research organisms

Policy information about studies involving animals; ARRIVE guidelines recommended for reporting animal research, and Sex and Gender in Research

Laboratory animals	Tdp43floxp/floxp (control) and Tdp43floxp/floxp; Zp3-Cre mice, Tdp43flox/flox;ER-cre mice and adult wild type ICR mice were used in our study.Tdp43floxp/floxp (control) and Tdp43floxp/floxp; Zp3-Cre mice, and Tdp43flox/flox;ER-cre mice were maintained in a hybrid background of C57BL/6J and ICR, Mice we used for experiment is adult about eight weeks, experimental and control animals were co-housed.
Wild animals	No wild animals were used in the study.
Reporting on sex	The mice used in our study were about eight weeks females, we investigate the function of maternal protein TDP-43 in oocyte to embryo transition, so we need to choose female mice for study.
Field-collected samples	No field collected samples were used in the study.
Ethics oversight	Mice are free for water and food supplies, and the mice are maintained and all experiments were performed under guidelines of the Animal Care and Use Committee of the Institute of Zoology, Chinese Academy of Sciences (IOZ-IACUC-2021-052).

Note that full information on the approval of the study protocol must also be provided in the manuscript.

Clinical data

Policy information about clinical studies

All manuscripts should comply with the ICMJE guidelines for publication of clinical research and a completed CONSORT checklist must be included with all submissions.

Clinical trial registration	
Study protocol	
Data collection	
Outcomes	

Dual use research of concern

Policy information about dual use research of concern

Hazards

Could the accidental, deliberate or reckless misuse of agents or technologies generated in the work, or the application of information presented in the manuscript, pose a threat to:

No	Yes	
☒	☐	Public health
☒	☐	National security
☒	☐	Crops and/or livestock
☒	☐	Ecosystems
☒	☐	Any other significant area

Experiments of concern

Does the work involve any of these experiments of concern:

No	Yes	
☒	☐	Demonstrate how to render a vaccine ineffective
☒	☐	Confer resistance to therapeutically useful antibiotics or antiviral agents
☒	☐	Enhance the virulence of a pathogen or render a nonpathogen virulent
☒	☐	Increase transmissibility of a pathogen
☒	☐	Alter the host range of a pathogen
☒	☐	Enable evasion of diagnostic/detection modalities
☒	☐	Enable the weaponization of a biological agent or toxin
☒	☐	Any other potentially harmful combination of experiments and agents

Plants

Seed stocks	None.
Novel plant genotypes	None.
Authentication	None.

ChIP-seq

Data deposition

☒ Confirm that both raw and final processed data have been deposited in a public database such as GEO.

☒ Confirm that you have deposited or provided access to graph files (e.g. BED files) for the called peaks.

Data access links
May remain private before publication.

https://www.ncbi.nlm.nih.gov/geo/query/acc.cgi?acc=GSE221985 token:yponqywutzervil

Files in database submission

Fastq and bigwig files of ChIP-seq data.
Stacc_TDP43_1C_rep1_r1.fq.gz
Stacc_TDP43_1C_rep1_r2.fq.gz
Stacc_TDP43_1C_rep2_r1.fq.gz
Stacc_TDP43_1C_rep2_r2.fq.gz
Stacc_TDP43_1C_rep3_r1.fq.gz
Stacc_TDP43_1C_rep3_r2.fq.gz
Stacc_TDP43_FGO_rep1_r1.fq.gz
Stacc_TDP43_FGO_rep1_r2.fq.gz
Stacc_TDP43_FGO_rep2_r1.fq.gz
Stacc_TDP43_FGO_rep2_r2.fq.gz
Stacc_TDP43_FGO_rep3_r1.fq.gz
Stacc_TDP43_FGO_rep3_r2.fq.gz
Stacc_TDP43_L2C_rep1_r1.fq.gz
Stacc_TDP43_L2C_rep1_r2.fq.gz

April 2023

Stacc_TDP43_L2C_rep2_r1.fq.gz
Stacc_TDP43_L2C_rep2_r2.fq.gz
Stacc_PolII_FGO_Ctrl_rep1_r1.fq.gz
Stacc_PolII_FGO_Ctrl_rep1_r2.fq.gz
Stacc_PolII_FGO_Ctrl_rep2_r1.fq.gz
Stacc_PolII_FGO_Ctrl_rep2_r2.fq.gz
Stacc_PolII_FGO_TDP43KO_rep1_r1.fq.gz
Stacc_PolII_FGO_TDP43KO_rep1_r2.fq.gz
Stacc_PolII_FGO_TDP43KO_rep2_r1.fq.gz
Stacc_PolII_FGO_TDP43KO_rep2_r2.fq.gz
Stacc_PolII_L2C_Ctrl_rep1_r1.fq.gz
Stacc_PolII_L2C_Ctrl_rep1_r2.fq.gz
Stacc_PolII_L2C_Ctrl_rep2_r1.fq.gz
Stacc_PolII_L2C_Ctrl_rep2_r2.fq.gz
Stacc_PolII_L2C_TDP43KO_rep1_r1.fq.gz
Stacc_PolII_L2C_TDP43KO_rep1_r2.fq.gz
Stacc_PolII_L2C_TDP43KO_rep2_r1.fq.gz
Stacc_PolII_L2C_TDP43KO_rep2_r2.fq.gz
Stacc_TDP43_1C_rep1.bw
Stacc_TDP43_1C_rep2.bw
Stacc_TDP43_1C_rep3.bw
Stacc_TDP43_FGO_rep1.bw
Stacc_TDP43_FGO_rep2.bw
Stacc_TDP43_FGO_rep3.bw
Stacc_TDP43_L2C_rep1.bw
Stacc_TDP43_L2C_rep2.bw
Stacc_PolII_FGO_Ctrl_rep1.bw
Stacc_PolII_FGO_Ctrl_rep2.bw
Stacc_PolII_FGO_TDP43KO_rep1.bw
Stacc_PolII_FGO_TDP43KO_rep2.bw
Stacc_PolII_L2C_Ctrl_rep1.bw
Stacc_PolII_L2C_Ctrl_rep2.bw
Stacc_PolII_L2C_TDP43KO_rep1.bw
Stacc_PolII_L2C_TDP43KO_rep2.bw

Genome browser session (e.g. UCSC)
None

Methodology

Replicates
2-3 replicates for Pol II Stacc-seq in WT and TDP KO FGOs or L2C embryos. 2-3 replicates for TDP43 Stacc-seq in WT FGOs and 1C/L2C embryos.

Sequencing depth
Varies in different Stacc-seq samples and can be checked at GEO accession GSE221985

Antibodies
anti-TDP-43: Proteintech 12892-1-AP,
anti-TDP-43: diagenode C15410266
anti-Pol II:Active Motif 102660

Peak calling parameters
MACS v1.4.2 with the parameters --nolambda --nomodel

Data quality
Reads with a Phred quality score of <20 were removed. Non-unique reads were removed by Samtools. Quality were accessed by UCSC Genome Browser.

Software
Bowtie2 v2.2.5, Samtools v1.3.1, MACS v1.4.2, ChIPseeker v1.26.2, BEDTools v2.29.2

Flow Cytometry

Plots

Confirm that:

☒ The axis labels state the marker and fluorochrome used (e.g. CD4-FITC).

☒ The axis scales are clearly visible. Include numbers along axes only for bottom left plot of group (a 'group' is an analysis of identical markers).

☒ All plots are contour plots with outliers or pseudocolor plots.

☒ A numerical value for number of cells or percentage (with statistics) is provided.

Methodology

Sample preparation

Instrument

Software

Cell population abundance

Gating strategy

☐ Tick this box to confirm that a figure exemplifying the gating strategy is provided in the Supplementary Information.

Magnetic resonance imaging

Experimental design

Design type

Design specifications

Behavioral performance measures

Acquisition

Imaging type(s)

Field strength

Sequence & imaging parameters

Area of acquisition

Diffusion MRI ☐ Used ☐ Not used

Preprocessing

Preprocessing software

Normalization

Normalization template

Noise and artifact removal

Volume censoring

Statistical modeling & inference

Model type and settings

Effect(s) tested

Specify type of analysis: ☐ Whole brain ☐ ROI-based ☐ Both

Statistic type for inference

(See Eklund et al. 2016)

Correction

Models & analysis

n/a	Involved in the study
☐	☐ Functional and/or effective connectivity
☐	☐ Graph analysis
☐	☐ Multivariate modeling or predictive analysis

Functional and/or effective connectivity

Graph analysis

Multivariate modeling and predictive analysis
